# Exploring the safety reporting culture among healthcare practitioners in Saudi hospitals: a comprehensive 2022 national study

**DOI:** 10.1186/s12913-024-11160-3

**Published:** 2024-06-28

**Authors:** Dyma Alkahf, Wadi Alonazi

**Affiliations:** Health Administration Department, College of Business Administration, King Saud University , PO Box 71115, Riyadh, 11587 Saudi Arabia

**Keywords:** Bed capacity, Descriptive study, Healthcare professionals, Patient safety culture, Reporting culture, Safety events, Staff position

## Abstract

**Background:**

With the rise in medical errors, establishing a strong safety culture and an effective incident reporting system is crucial. As part of the Saudi National Health Transformation Vision of 2030, multiple projects have been initiated to periodically assess healthcare quality measures and ensure a commitment to continuous improvement. Among these is the Hospital Survey on Patient Safety Culture National Project (HSPSC), conducted regularly by the Saudi Patient Safety Center (SPSC). However, comprehensive tools for assessing reporting culture are lacking. Addressing this gap can enhance reporting, efficiency, and health safety.

**Objective:**

This paper aims to investigate the reporting practices among healthcare professionals (HCPs) in Saudi Arabian hospitals and examine the relationship between reporting culture domains and other variables such as hospital bed capabilities and HCPs’ work positions.

**Methods:**

The study focuses on measuring the reporting culture-related items measures and employs secondary data analysis using information from the Hospital Survey on Patient Safety Culture conducted by the Saudi Center for Patient Safety in 2022, encompassing hospitals throughout Saudi Arabia. Data incorporated seven items in total: four items related to the Response to Error Domain, two related to the Reporting Patient Safety Events Domain, and one associated with the number of events reported in the past 12 months.

**Results:**

The sample for the analyzed data included 145,657 HCPs from 392 hospitals. The results showed that the average positive response rates for reporting culture-related items were between 50% and 70%. In addition, the research indicated that favorable response rates were relatively higher among managerial and quality/patient safety/risk management staff. In contrast, almost half had not reported any events in the preceding year, and a quarter reported only 1 or 2 events. Pearson correlation analysis demonstrates a strong negative correlation between bed capacity and reporting safety events, response to error, and number of events reported (*r* = -0.935, -0.920, and − 0.911, respectively; *p* < 0.05), while a strong positive correlation is observed between reporting safety events and response to error (*r* = 0.980; *p* < 0.01).

**Conclusions:**

Almost 75% of the HCPs reported fewer safety events over the last 12 months, indicating an unexpectedly minimal recorded occurrence variance ranging from 0 to 2 incidents.

## Background

Patient safety aims to avoid harm [1]. In the USA, medical errors are ranked as the third cause of death [2]. Saudi Arabia has also witnessed an increase in healthcare-related court complaints, with 6,631 cases in 2021 and 7,698 cases in 2022. The number of resolved malpractice death cases was 517 in 2021 and 597 in 2022, with conviction rates of 33% and 29%, respectively [3, 4]. Thus, establishing a strong safety culture and an effective incident reporting system is a priority.

The “safety culture” is the shared values, attitudes, and behaviors that shape an organization’s commitment to patient safety [1]. That concept emerged in healthcare after the report “To Err is Human” by the Institution of Medicine (IOM) in 1999, which revealed that preventable medical errors lead to thousands of deaths annually in the U.S [5, 6]. . This is in agreement with “Crossing the Quality Chasm” report for 2001 which emphasized the need to establish an error-detecting and learning commitment initiatives [7, 8]. Safety culture comprises five dimensions: Informed, Learning, Just, Flexible, and Reporting Cultures. These dimensions ensure clear safety information, adaptability, accountability without fear of reprisal, effective management of safety issues, and open and honest reporting [9]. Small slips, accidental violations, and even close calls escape reporting, potentially eliminating valuable proactive safety and quality improvement prospects [10, 11]. Thus, continuous assessment and monitoring to guarantee reporting culture is important.

The Survey on Patient Safety Culture (SOPS) by the Agency for Healthcare Research and Quality (AHRQ) is the most widely used tool [12]. It comprises ten domains, two of them are directly related to reporting culture, the Response to Error Domain and the Reporting Patient Safety Events Domain [13]. The National Transformation Vision 2030 in Saudi Arabia led to the establishment of the Saudi Patient Safety Center (SPSC) in 2017. It annually assesses hospital safety culture using SOPS tool [14, 15].

Many studies have assessed safety culture internationally in health institutions. Kim et al. found that most nurses in a Korean hospital did not feel comfortable reporting errors or communicating safety issues. They recommended building non-punitive environment [16]. Paradiso et al. examined the correlation between just culture, trust, and error reporting, finding significant differences in perceptions between nurse managers and clinical nurses [17]. Ranaei et al. studied Iran’s medical error-reporting system, recommending workshops to increase the error-reporting rate [18].

In Saudi hospitals, studies about patient safety culture is mostly focuses on the perception of the healthcare workers. Albalawi et al., for example, conducted a study describing the challenges faced regarding the development of effective patient safety culture, including leadership issues, the blame culture, inadequate staffing, and communication challenges. They acknowledged factors such as supportive leadership, collaborative team work, and making continuous improvement [19, 20]. Alrasheadi et al. was aimed on the relationship between safety culture, leadership and medication error reporting of the nurses [21]. Binkheder and others examined the relation between patient safety culture domains and sentinel events [14].

Currently, there is a lack of research focusing on comprehensive tool that can be employed in assessing the Saudi hospitals reporting culture rather than safety culture as whole. Addressing this gap will also contribute to enhance reporting by defining the opportunities for best practice. A properly implemented system of reporting incidents can minimize the administrative tasks, enhance efficiency, and mitigate the risk to people’s health and lives. Thus, the research aims to explore the reporting culture among HCPs in Saudi Arabia and study the relationship between reporting culture and variables like hospital bed capacity and staff positions.

## Methods

### Study design

The study employes a descriptive and analytical design.

### Study data setting and data sources

The study utilized secondary data, the Hospital Survey on Patient Safety Culture (HSPSC), collected nationally in Saudi Arabia from the Saudi Patient Safety Center (SPSC) and available online. Data were collected between January and March 2022 [22]. The sampling encompassed all responses from the Saudi Patient Safety Center  (SPSC), indicating a comprehensive and inclusive approach. This methodology helps mitigate sampling biases and ensures a broader perspective on the issues under investigation.

### Dataset, hospital survey on patient safety culture (HSPSC)

The HSPSC utilizes the Survey on Patient Safety Culture (SOPS) tool by AHRQ, a validated and internationally used tool for assessing hospital safety culture. The latest version is version 2.0, which has 32 items that tally ten domain measures. Also, there are two extra items: the number of events reported in the past 12 months and the overall patient safety rating [11].

Seven of the 34-item measures will be studied and correlated as they are explicitly related to the institution’s reporting culture. There are four items related to the Response to Error Domain, two related to the Reporting Patient Safety Events Domain, and one about the number of events reported in the past 12 months. Since they are secondary data, the average percentage (%) of positive responses to the items was examined.

Furthermore, demographic data were gathered, encompassing the bed capacity, staff positions, work areas, and weekly working hours.

The items or questions were scored using either a frequency scale (1 = “never” to 5 = “always”) or a five-point Likert scale of agreement (1 = “strongly disagree” to 5 = “strongly agree”). They used the percent positive per hospital for these patient safety culture domains for the calculation. Every item measure has a potential range of 0–100% positive. Negatively phrased items were reverse-coded before analysis, meaning higher scores indicate positive replies. The mean percentage of positive scores for the constituent items within each domain measure was calculated to determine each domain’s overall rate of positive scores. When asked, “In the past 12 months, how many event reports have you filled out and submitted?” they counted “1 or more event reports” as a positive response answer [12].

#### Outcomes and exploratory variables

Seven item measures express the reporting culture. There are four items related to the Response to Error Domain, two related to the Reporting Patient Safety Events Domain, and one about the number of events reported in the past 12 months. The % favorable response rates for these item measures were correlated with other variables like hospital bed capabilities and HCPs’ work positions.

#### Statistical analysis tools

The tools utilized for data analysis, comparison, and visualization were IBM SPSS Statistics for Windows version 26 and Microsoft Excel 2016. Descriptive statistics were used to compute frequencies and percentages. The relationships between the variables were measured by applying Pearson correlation in bivariate analysis. Linear regression was applied to find a statistically significant domain predictor for the dependent variable.

## Results

### Demographic characteristics of respondents

The final dataset included 145,657 staff responses from 392 hospitals. The respondents were from different healthcare sectors and working positions. As shown in Table 1 below, 59.1% of respondents worked in Ministry of Health (MOH) hospitals. The highest percentage of respondents (23.3%) were working in hospital bed capacity, more than 501. Most staff are nurses 47.2%, physicians 19.5%, and technologists 6.4%.

The average hospital response rate during this cycle was 70.2%, ranging from 3.7 to 100%, and the average number of respondents per hospital was 372, ranging from 16 to 5,245. Most respondents directly interact with patients (84.3%), while the rest do not. The working hours per week ranged from less than 30 h (3.1%), 30 to 40 h (27.9%), and more than 40 h (69.1%).

**Table 1:** Demographic characteristics of respondents

Hospital Sector	Respondents % (*n* = 145,657)		Hospital Bed Capacity		Respondents % (*n* = 145,657)
MOH	59.1		50–100		22.1
Governmental non-MOH	25.0		101–200		18.4
Private	15.9		201–300		14.3
			301–500		22.0
			501 and more		23.3
**Staff Positions**					**Respondents % (** ***n*** ** = 145,657)**
Nurse					47.2
Physician					19.5
Technologists like EKG, ECMO, Neuro., Catheterization, Lab, Radiology					6.4
Managers					4.2
Pharmacist					3.8
Physical, Occupational, Prosthetics, Speech therapist					2.1
Unit Clerk, Secretary, Receptionist					2.9
Patient Experience, Patient Relation, Bed, and Case Management					1.3
Paramedics					1.2
Quality, Patient Safety, Risk Manager, Clinical Audit, Performance Improvement					1.2
Respiratory Therapist					1.2
Dietician					1.1
Social Workers					1.1
Healthcare Assistant					0.9
Infection Control					0.9
Others					5.1

### Reporting culture-related item measures results

The study explored the measures that indicate the culture of reporting. These include the Response to Error Domain, Reporting Patient Safety Events Domain, each composed of 1 to 4 questions or items, and the Number of Events Reported last year.

**Table 2:** The average % positive response for selected HSPSC item measures

HSPSC Item Measures	Average % Positive Response	
**Response to Error Domain**		
A6. In this unit, staff feel like their mistakes are held against them*		51.3
A7. When an event is reported in this unit, it feels like the person is being written up, not the problem*		49.7
A10. When staff make errors, this unit focuses on learning rather than blaming individuals		70.0
A13. In this unit, there is a lack of support for staff involved in patient safety errors*		51.4
**Reporting Patient Safety Events Domain**		
D1. When a mistake is caught and corrected before reaching the patient, how often is this reported?		65.5
D2. When a mistake reaches the patient and could have harmed the patient but did not. How often is this reported?		67.7
**Number of Events Reported**		
D3. 1 or more events reported in the last 12 months		51.7

* Negatively worded items were reverse-coded so that higher scores represent positive responses

Table 2 describes that the Response to Error Domain is made of four item measures. The highest percentage is (70.0%) to A10, which means that the department usually prefers to do something to learn from their mistakes rather than blaming someone in particular. On the contrary, the smallest positive reaction rate (49.7%) belongs to A7, which is reported as pointing at the tendency to blame staff who are surrounded by errors and not looking at how actual issues can be addressed in a crisis.

Conversely, the Reporting Patient Safety Events Domain has entitled two item measures. Considering that D1 was 65.5% and D2 was 67.7% meaning that reporting near misses and no-harm errors happened frequently.

**Table 3:** The average % positive response for different staff working positions

Staff Positions	Response to Error	Reporting Events
Nurse	53.0	64.2
Physician	57.0	62.4
Technologist	56.1	63.3
Managers	67.5	72.5
Pharmacist	60.3	74.7
Physical/Occupational/Prosthetics/Speech therapist	60.2	63.5
Unit Clerk/Secretary/Receptionist	54.7	61.9
Patient Experience/Patient Relation/Bed and Case Management	56.3	62.5
Paramedics	50.8	60.4
Quality/Patient Safety/Risk Manager/Clinical Audit/Performance Improvement	69.7	71.0
Respiratory Therapist	54.8	61.0
Dietician	63.2	67.4
Social Workers	60.1	63.0
Healthcare Assistant	50.9	62.4
Infection Control	62.3	70.5
Others	56.9	66.1

The job titles of the respondents were quite diverse, as shown in Table 3. Regarding the Response to Error Domain, the highest percentages of favorable responses were 69.7% for roles connected with Quality/Patient Safety/Risk Manager/Clinical Audit/Performance Improvement and 67.5% for managers. They seem more likely to see errors as a chance for learning. Comparatively, the percentages of positive responses in Healthcare Assistants (50.9%) and Paramedics (50.8%) were lower than those in other roles.

However, when it comes to the Reporting Patient Safety Events Domain, the most significant percentages were from pharmacists (74.7%), managers (72.5%), and infection control areas (70.5%). Roles such as these may be more active when reporting incidents than Unit Clerk/Secretary/Receptionist and Healthcare Assistant roles, which have smaller percentages (61.9% and 62.4%, respectively) in reporting safety occurrences.

### Reporting culture-related item measures results linked with hospital bed capacity

Hospitals were categorized based on the number of beds. The average percentage of the positive response rate was calculated separately for each hospital bed capacity category.

**Fig. 1:**
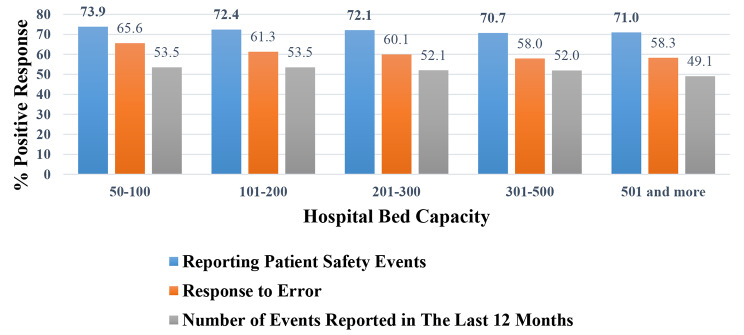
The % positive response for reporting culture item measures linked with bed capacity

As shown in Fig. 1, the highest percentage of positive responses is in the Reporting Patient Safety Events Domain, with smaller bed capacity units (50–100 beds) feeling better about reporting than more extensive facilities (500 and more beds). However, the differences are minor. As bed capacity increases, the response to the error domain and the number of events reported show a steady decrease in positive responses.

### The number of events reported in the last 12 months

**Fig. 2:**
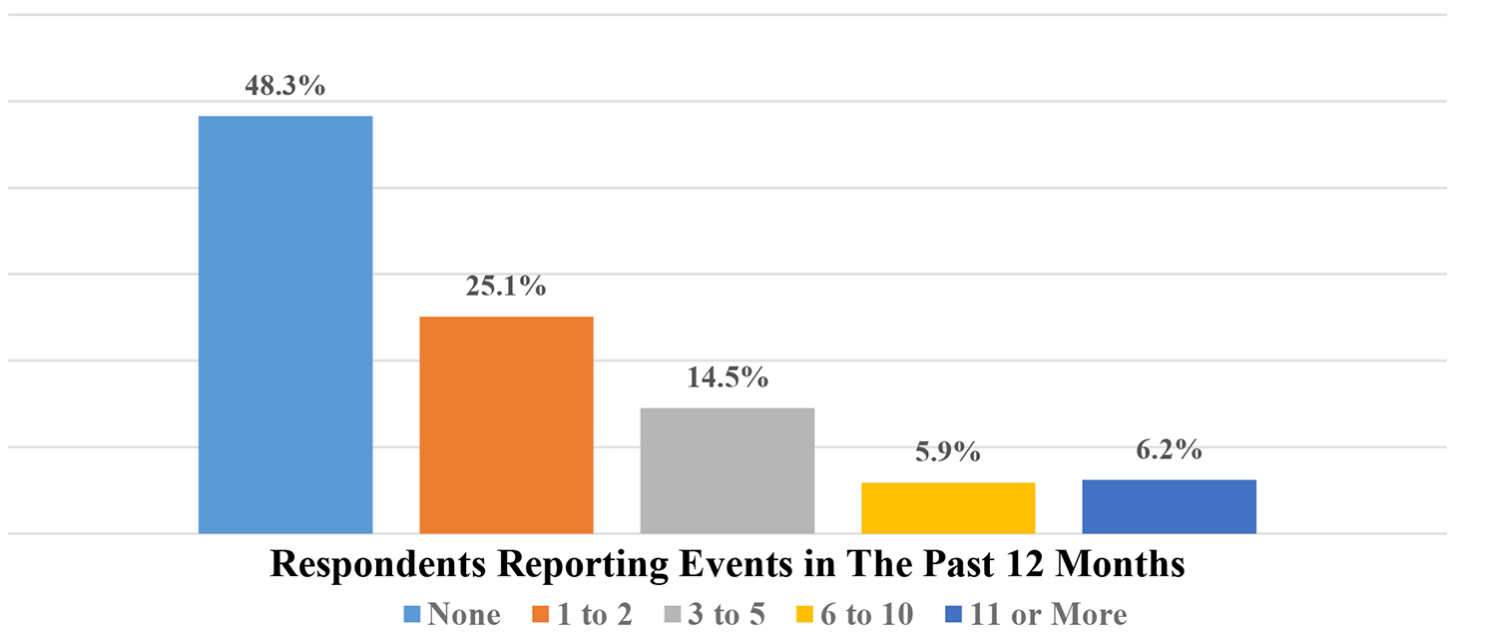
Number of events reported in the past 12 months

How many incidents were reported by staff during the past 12 months is shown in Fig. 2. 48.3% of the respondents did not report any events, while 25.1% only reported one or two incidents in the previous year. This shows that many respondents encountered and reported a limited number of events yearly, while fewer reported higher counts of events.

### Relationship between reporting culture item measures and hospital bed capacity

A Pearson correlation analysis by SPSS was conducted between hospital bed capacity, % positive response rate for Reporting Safety Events Domain, Response to Error Domain, and Number of Events Reported. The analyzed data were explored previously in Fig. 1.

**Table 4:** The correlation between hospitals’ bed capacity and % positive response of reporting culture item measures

		Bed Capacity	Reporting Safety Events	Response to Error	No. of Events Reported
**Bed Capacity**	Pearson Correlation	1	− 0.935^*^	− 0.920^*^	− 0.911^*^
	Sig. (2-tailed)		0.020	0.027	0.031
	N	5	5	5	5
**Reporting Safety Events**	Pearson Correlation	− 0.935^*^	1	0.980^**^	0.709
	Sig. (2-tailed)	0.020		0.003	0.180
	N	5	5	5	5
**Response to Error**	Pearson Correlation	− 0.920^*^	0.980^**^	1	0.691
	Sig. (2-tailed)	0.027	0.003		0.196
	N	5	5	5	5
**No. of Events Reported**	Pearson Correlation	− 0.911^*^	0.709	0.691	1
	Sig. (2-tailed)	0.031	0.180	0.196	
	N	5	5	5	5

* Correlation is significant at the 0.05 level (2-tailed).

** Correlation is significant at the 0.01 level (2-tailed).

As shown in Table 4, the correlation of Bed Capacity versus Reporting Safety Events, Response to Error, and Number of Events Reported was strongly negative according to the analysis with *r* = − 0.935, -0.920, and − 0.911, respectively. This correlation is statistically significant at the 0.05 level (2-tailed) and likely did not happen by chance. Therefore, it means that as the bed capacity increases, there may be a trend of a decrease in the number of reported events, perceived error response, and safety reporting.

On the other hand, Reporting Safety Events versus Response to Error and Number of Events Reported demonstrate a robust positive correlation with *r* = 0.980 (*p* < 0.01) and 0.709, respectively. This means that as reporting safety events goes up, there is an increase in a positive response to errors and the number of reported incidents.

Furthermore, when there is a better response to mistakes, the count of noted events seems to rise. This is shown by the moderately significant correlation (*r* = 0.691) between Response to Error and Number of Events Reported.

### Reporting culture domains measures across various staff work positions

A Pearson correlation analysis by SPSS was conducted between the % positive response rate for the Reporting Safety Events Domain and Response to Error Domain across different staff positions. The analyzed data were explored previously in Table 3.

According to Pearson Correlation scores, Table 5 shows a strong positive association (*r* = 0.766) between the Response to Error Domain and the Reporting Patient Safety Events Domain. This connection is statistically significant at the 0.01 level (2-tailed). Besides, we studied the link between the dependent variable, Reporting Patient Safety Events, and the independent variable, Response to Error, using the linear regression analysis method.

**Table 5:** The correlation between response to error and reporting patient safety events domains results across various staff positions

		Response to Error	Reporting Patient Safety Events
**Response to Error**	Pearson Correlation	1	0.766^**^
	Sig. (2-tailed)		0.001
	N	16	16
**Reporting Patient Safety Events**	Pearson Correlation	0.766^**^	1
	Sig. (2-tailed)	0.001	
	N	16	16

** Correlation is significant at the 0.01 level (2-tailed).

**Table 6:** The regression model of the study

Regression Model Summary									
Model	*R*	*R* Square		Adjusted *R* Square			Std. Error of the Estimate		
1	.766^a^	0.586		0.557			2.964		
a. Predictors: (Constant), Response to Error									
**ANOVA** ^**a**^									
**Model**	**Sum of Squares**		**df**	**Mean Square**		**F**		**Sig.**	
Regression	174.306		1	174.306		19.844		.001^b^	
Residual	122.977		14	8.784					
Total	297.283		15						
a. Dependent Variable: Reporting Patient Safety Events									
b. Predictors: (Constant), Response to Error									
**Coefficients** ^**a**^									
**Model**	**Unstandardized Coefficients**				**Standardized Coefficients**			**t**	**Sig.**
	**B**		**Std. Error**		**Beta**				
Constant	28.816		8.253					3.492	0.004
Response to Error	0.627		0.141		0.766			4.455	0.001
a. Dependent Variable: Reporting Patient Safety Events									

As shown in Table 6, our formula’s Response to Error factor suggests it is responsible for around 58.6% of changes in the Reporting Patient Safety Events Domain. The adjusted R Square value indicates that almost 55.7% of changes are explained when we adjust for different variables. The standard error measurement, 2.964, shows how far our real results deviate from our predictions, giving us a sense of accuracy.

A *p*-value of.001 in the ANOVA table signals that our model is statistically significant. In simpler terms, the model we have made helps us predict patient safety events reporting based on how errors are handled.

The intercept of 28.816 suggests that if there is no engagement with errors, we estimate 28.816 reports on patient safety events. Add onto this a slope of 0.627. It says as we improve response to an error by one step, we expect patient safety reports to increase by 0.627. Let us also ponder over the *t*-value of 4.455 and *p*-value of 0.001 for error response. These values confirm how errors are made and confirm that they are crucial in measuring the number of patient safety reports.

## Discussion

In the 2022 Hospital survey on patient safety culture, the seven items related to the reporting culture were examined and correlated. The study found that the overall favorable response rates for the reporting culture-related item measures are around 50–70%, which shows differing views about error correction, staff help, and reporting practice.

This analysis of reporting culture domains suggests a potential for improvement, especially in changing the perception around event reporting to emphasize problem-solving and solution-generating rather than individual blame [23]. Also, it emphasizes the importance of providing better support for the second victim, the term first used by Albert Wu to describe the staff who was involved in the error [24]. Also, maintaining or improving the positive aspects, such as learning from mistakes, could further strengthen the patient safety culture within the unit [25].

Managers and individuals within quality, patient safety, and risk management roles seem to score higher on average, positively and proactively, in regard to mistakes and safety-related incidents. This shows a culture concentrated on learning from past mistakes and actively reporting events. On the other hand, Healthcare Assistants and paramedics have lower scores in both two domains. This identifies areas that might need enhancement in their reporting culture. Activities such as specifically designed training sessions or interventions centered on error detection and safety incident reporting could help roles with lower scores [26], cultivating a culture for learning and proactive reporting [27]. Acknowledging and spreading effective approaches from roles with higher scores might assist in irradiating positive conduct throughout the institution and promoting a robust patient safety environment for all roles [28].

The research indicates that the smaller hospitals have higher positive response ratings. When hospitals grow bigger, problems may arise. This could relate to communication, processes, or how much work there is. These studies indicate that hospital size could impact how patient safety is viewed. This is especially correct for error handling and reporting. Larger hospitals may need more specialized techniques to enhance responses to errors and encourage reporting. Tailored training lectures, creating a safety culture, allocating initiatives, or adjusting the reporting systems could benefit these larger health institutions.

By studying the number of events reported by staff in the past year, it showed that 1/2 of the staff had not reported any event, and 1/4 reported just 1 or 2 events. The prominent underreporting tendency warrants investigation to reveal the potential barriers to reporting patient safety events among staff. Also, that may negatively affect the accuracy and validity of reporting culture-associated statistics. In addition, it might influence the need to design and implement interventions to encourage reporting across all staff. Reporting promotion could contribute to a more systemic understanding of safety issues within the organization [29]. Addressing possible reporting obstacles like workload, shortage of team of workers, worry of disciplinary action, lack of prompt feedback and solutions, and difficult-to-use reporting system [30]. Also, promoting a reporting culture and enforcing a robust and thorough incident reporting system within the organization might lead to better incident catch-and-follow improvement results.

The correlation analysis between variables showed that bigger hospitals are linked with less reporting of safety events, poorer responses to errors, and fewer events reported last year. Also, there is a positive relationship between reporting safety events, positive response to errors, and the number of events reported, indicating that as reporting increases, the positive response and the actual reporting of events is increased accordingly.

The regression analysis demonstrated a strong positive linear relationship between Response to Error and Reporting Patient Safety Events Domains scores (*r* = 0.766). This guarantees that units or environments with a more positive attitude toward dealing with errors tend to have higher rates of reporting patient safety incidents and vice versa. The hypothesis behind the regression model indicates that a culture of learning from mistakes or errors often coincides with a greater tendency to report safety-related incidents or events. Institutions that encourage enhancing patient safety culture must fully understand this high positive correlation. Reporting patient safety events can be improved if the proper response to errors is generated in a positive light. Also, a greater tendency to disclose safety occurrences resulted in a more effective response to errors.

The Linear Regression Equation using Pearson’s correlation coefficient can be concluded as **ŷ = b0 + b x = 28.816 + 0.627 x** (where **ŷ** is the predicted % positive score of Reporting Patient Safety Events Domain, **x** is the % positive score of Response to Error Domain, **b0** is the y-intercept or the value of y when x = 0, and **b** is the slope or how y changes per unit increase in x). That equation is useful to forecast and anticipate the Reporting Patient Safety Events Domain in a given Response to Error Domain score.

### Strengths and limitations of study

The data set is on a Saudi national level, and the number of respondents is high, leading to more relevant results and conclusions. As more in-depth analysis requires the availability of the row data, the primary data were not published, so the averages of measures were used in the study. The practical implications of this research highlight the importance of decision-makers addressing the reporting culture within large hospitals. The findings suggest the need for implementing new approaches to address the concerns related to reporting practices effectively.

## Conclusion

This research focused on exploring the reporting culture among staff in Saudi hospitals. The overall favorable response rates for the reporting culture-related domains are around 50–70%, and half of the staff have not reported any events in the past year. That implied a mix of perceptions regarding error handling and reporting. Managers and Quality/Patient Safety/Risk Management positions were at the top of the reporting culture. The analysis discovered statistically significant relationships between the variables. It found a strong negative relationship between hospital bed capacity and reporting culture. A strong positive correlation was found between the reporting cultures domains. This research is fundamental to further investigating the barriers that prevent staff from reporting near misses and events and to find solutions for the obstacles addressed. Furthermore, it suggests a potential for improvement by encouraging reporting through implementing just cultural principles, giving incentives to staff, developing training programs on patient safety, and creating supporting programs for the second victims.

## Data Availability

The datasets analyzed during the current study are available on the Saudi Patient Safety Center’s website, https://www.spsc.gov.sa/English/HSPSC/Pages/national-report.aspx.

## Abbreviations

HSPSC: Hospital Survey on Patient Safety Culture
SPSC: Saudi Patient Safety Center
HCPs: Healthcare Professionals
USA: United States of America
IOM: Institution of Medicine
SOPS: Hospital Survey on Patient Safety Culture
AHRQ: Agency for Healthcare Research and Quality
MOH: Ministry of Health
